# The Association Between Kinesiophobia and Level of Mobilization in Patients After Open-Heart Surgery

**DOI:** 10.3390/healthcare14101334

**Published:** 2026-05-13

**Authors:** Aleyna Tufan, Gizem Kubat Bakir

**Affiliations:** 1 Koşuyolu High Specialty Training and Research Hospital, T.R. Ministry of Health, Istanbul 34903, Türkiye; aleyna-tufan@hotmail.com; 2 School of Nursing, Maltepe University, Istanbul 34857, Türkiye

**Keywords:** kinesiophobia, mobilization, open-heart surgery, postoperative recovery, nursing care, surgical nursing, cross-sectional study

## Abstract

**Background/Objectives:** Early mobilization following open-heart surgery is a key component of postoperative recovery, yet psychological barriers such as kinesiophobia (fear of movement) may limit patient participation. This study examined the association between kinesiophobia and mobilization level in patients after open-heart surgery and explored sociodemographic and clinical correlates of both variables. **Methods:** A cross-sectional descriptive design was used. The sample comprised 96 adult cardiac surgery patients recruited consecutively from cardiovascular surgery ICUs at two centers in Istanbul—a public training and research hospital and a foundation-affiliated university hospital—between December 2024 and April 2025. Data were collected via a Personal Information Form, the Tampa Scale of Kinesiophobia (TSK), and the Intensive Care Units Mobility Scale (IMS). Analyses (SPSS 25.0) included Mann–Whitney U and Kruskal–Wallis H tests, Pearson correlation with 95% confidence intervals (CIs) calculated via Fisher’s z-transformation, Bonferroni correction for k = 12 subgroup comparisons within each outcome, and a multivariable linear regression adjusted for sex, age, smoking, and history of surgery. **Results:** Of the 96 patients enrolled, 76.0% were male, with a mean age of 58.30 ± 6.50 years (SD) and a mean body mass index of 27.53 ± 5.84 kg/m^2^. The mean TSK total score was 46.81 ± 6.51 and the mean IMS score was 5.48 ± 0.73. Kinesiophobia and mobilization showed a small inverse association that reached statistical significance (r = −0.104; 95% CI: −0.298 to 0.099; r^2^ = 0.011; *p* = 0.041), accounting for approximately 1% of the variance in mobilization. After multivariable adjustment, kinesiophobia was no longer a significant predictor (β = −0.092; *p* = 0.360), whereas smoking (β = −0.279; *p* = 0.008) and female sex (β = 0.215; *p* = 0.039) emerged as the strongest independent correlates. Mobilization level differed by gender and smoking, and kinesiophobia level differed by marital status, history of surgery, and family history of heart disease at the uncorrected level; however, none of these subgroup differences remained significant after Bonferroni correction. **Conclusions:** Higher kinesiophobia scores were associated with lower mobilization levels following open-heart surgery, but the effect size was small and the association did not persist after adjustment for clinical confounders. The cross-sectional design precludes causal inference. Kinesiophobia may be considered as one of several psychosocial factors potentially relevant to postoperative mobilization rather than as a primary determinant.

## 1. Introduction

Cardiovascular diseases continue to represent a major contributor to global mortality and morbidity, despite considerable progress in diagnostic methods and therapeutic options across both high-income and low- and middle-income settings. Approximately 9 million people die each year globally due to cardiovascular diseases [1]. According to the 2022 mortality statistics of the Turkish Statistical Institute (TÜİK), a total of 504,839 deaths occurred in Türkiye, of which 35.4% were attributable to diseases of the circulatory system. Among these, ischemic heart diseases accounted for 42.3%, other heart diseases for 23.5%, and cerebrovascular diseases for 19.2%. Cardiovascular diseases therefore continue to constitute a major public health problem and a significant burden on the healthcare system in Türkiye. This burden underscores the necessity of developing effective rehabilitation programs and evidence-based nursing care strategies for the prevention, treatment, and recovery of these conditions [2].

Among available therapeutic options for cardiovascular conditions, open-heart surgery is one of the most established interventions, supported by evidence of clinical efficacy and an acceptable safety profile [3,4,5]. While the procedure offers important gains in survival and overall quality of life, it simultaneously imposes substantial physiological demands on several body systems—most notably cardiovascular, pulmonary, and neurological functions [6]. Due to these multifaceted physiological effects, patients require close monitoring and carefully planned care processes in the postoperative period. Patients are followed in the intensive care unit after surgery, where hemodynamic parameters and respiratory functions are regularly assessed [6,7]. Once spontaneous respiration reaches an adequate level and hemodynamic stability is achieved, extubation is performed; following extubation, level of consciousness, pain intensity, and cardiopulmonary functions are meticulously monitored by nurses [7].

Clinical stability achieved during the intensive care process enables the initiation of early mobilization, which is a critical component of postoperative recovery. Early mobilization after open-heart surgery is considered a fundamental nursing intervention for preventing complications, supporting respiratory and circulatory functions, preserving muscle strength, and maintaining psychological well-being [7]. When hemodynamic stability is ensured, the mobilization process progresses gradually through positioning in a sitting posture, sitting at the edge of the bed, transferring to a chair, and standing. Early mobilization has been shown to increase respiratory capacity, improve cardiac output and venous return, and support myocardial function by reducing myocardial oxygen consumption [8]. It also reduces the incidence of complications associated with prolonged bed rest, such as atelectasis, pneumonia, deep vein thrombosis, and muscle weakness [9].

Despite the control of physiological factors that may clinically hinder mobilization, some patients are reluctant to participate in the mobilization process and exhibit avoidance behaviors toward movement. In the postoperative period, mobilization may be complicated by physical and psychological factors such as pain, weakness, fatigue, sedation, nausea, and orthostatic instability. In addition to these factors, kinesiophobia (fear of movement) emerges as a significant barrier. Kinesiophobia is defined as the avoidance of movement due to the belief that activity will increase pain or worsen the disease. This fear may be associated with reduced participation in mobilization and with poorer functional outcomes [10,11].

In individuals experiencing kinesiophobia during the postoperative period, reduced participation in activity may prolong the rehabilitation process and increase the risk of complications. Nurses should therefore consider not only patients’ physical status but also their cognitive and emotional responses related to movement. Early identification of kinesiophobia and the provision of appropriate education and motivation may enhance the effectiveness of mobilization and reduce the risk of complications [12,13].

Although the physiological benefits of early mobilization after open-heart surgery have been well documented in the literature, psychological determinants of mobilization, particularly kinesiophobia, have been addressed in a limited number of studies. Kinesiophobia has primarily been investigated in the context of musculoskeletal disorders, chronic pain syndromes, or orthopedic surgery. However, its association with mobilization level in the postoperative cardiac surgery period, especially under intensive care conditions, has not been sufficiently explored. Moreover, most existing studies either focus solely on physical mobilization outcomes or treat kinesiophobia as a secondary variable, without comprehensively evaluating the direct association between these two concepts. While prior studies have separately examined either physical or psychological determinants of postoperative mobilization, no study to date has quantitatively assessed the direct association between kinesiophobia and ICU-level mobilization in cardiac surgery patients using validated instruments. The present study addresses this specific gap and highlights the possibility that mobilization in patients undergoing open-heart surgery may be associated not only with physiological but also with psychological barriers.

This study therefore aims to examine the association between kinesiophobia and mobilization level in patients who have undergone open-heart surgery. Demonstrating such an association may inform postoperative care by supporting the systematic assessment of patients’ movement-related fears alongside hemodynamic and respiratory stability. The findings may help nurses identify kinesiophobia at an early stage, plan individualized education and counseling interventions, and adapt mobilization programs to patients’ psychological readiness. The results are expected to contribute to the clinical literature on early mobilization, complication prevention, and recovery after open-heart surgery.

Based on prior literature suggesting that fear of movement may interfere with postoperative recovery, we hypothesized that higher kinesiophobia scores would be associated with lower mobilization levels in patients undergoing open-heart surgery. The formal hypothesis statement is provided in the Methods section.

## 2. Materials and Methods

### 2.1. Study Design

This study was conducted as a cross-sectional, single-time-point descriptive study to determine the association between kinesiophobia and mobilization level in patients who underwent open-heart surgery. Given the cross-sectional nature of the design, the study was intended to identify associations rather than to infer temporality, directionality, or causality between kinesiophobia and mobilization.

### 2.2. Aim of the Study, Research Questions, and Hypothesis

The aim of this study was to identify the association between kinesiophobia and mobilization level in patients who underwent open-heart surgery. The research sought to answer the following questions:

1. What are the sociodemographic and health history characteristics of patients who underwent open-heart surgery?
2. What are the total mean scores of the Tampa Scale of Kinesiophobia and the Intensive Care Units Mobility Scale in patients who underwent open-heart surgery?
3. Is there a significant association between kinesiophobia and mobilization level in patients who underwent open-heart surgery?
4. Is there a significant association between the sociodemographic characteristics of patients who underwent open-heart surgery and their Tampa Scale of Kinesiophobia and Intensive Care Units Mobility Scale scores?

Primary hypothesis (confirmatory): Based on prior literature suggesting that fear of movement may interfere with postoperative recovery, we hypothesized that higher kinesiophobia scores would be associated with lower mobilization levels in patients undergoing open-heart surgery, with an expected negative direction of association. Subgroup comparisons across sociodemographic and clinical variables (Research Question 4) were considered exploratory rather than confirmatory.

### 2.3. Setting and Time of the Study

The study was conducted between December 2024 and April 2025 with patients receiving treatment in the cardiovascular surgery intensive care units of a public training and research hospital and a medical faculty affiliated training and research hospital of a foundation university in Istanbul.

### 2.4. Population and Sample

The target population comprised adult cardiac surgery recipients receiving postoperative care in the cardiovascular surgery ICUs of two Istanbul-based institutions: a public training and research hospital and a foundation-affiliated university hospital. A total of 96 patients who met the study criteria between December 2024 and April 2025 were included in the sample.

An a priori power calculation was performed in G*Power 3.1.9.4 (Heinrich Heine University, Düsseldorf, Germany) under the following parameters: two-tailed α of 0.05, statistical power of 0.80, and a moderate Cohen’s d of 0.50. This yielded a target enrollment of 87 participants. To account for the possibility of non-parametric testing, the sample size was increased by 10%, and the study was conducted with 96 patients. The sample size was calculated for correlation analysis. Patients were recruited using a consecutive sampling method. Mobilization level was assessed during the first postoperative mobilization session in the ICU once the patient was hemodynamically stable, extubated, and oriented; this typically occurred within the first 24–48 h after surgery, although the exact postoperative day varied across patients depending on individual clinical recovery (e.g., extubation timing, hemodynamic stabilization). All assessments were therefore conducted at a clinically comparable milestone—the first mobilization session—rather than on a fixed postoperative day.

A post hoc consideration of statistical power should be acknowledged: the a priori calculation was based on a medium effect size (d = 0.50, equivalent to |r| ≈ 0.24); however, the observed correlation in this study (|r| = 0.104) corresponds to a much smaller effect size. Detecting a correlation of |r| = 0.10 with 80% power and α = 0.05 would require approximately 783 participants. The present study is therefore underpowered to confirm small effects with confidence; this limitation is acknowledged in the Discussion.

Eligibility was determined according to the following criteria. Inclusion criteria: (i) ≥18 years of age; (ii) status post open-heart surgical procedure; (iii) no requirement for inotropic infusion at the time of assessment; (iv) absence of any contraindication to mobilization; and (v) voluntary written consent to participate. Exclusion criteria: (i) age below 18; (ii) documented psychiatric diagnosis; (iii) ongoing inotropic support; (iv) clinical contraindication to bedside mobilization; or (v) declining to participate.

### 2.5. Data Collection Tools and Procedures

The study data were collected using the Personal Information Form, the Intensive Care Units Mobility Scale, and the Tampa Scale of Kinesiophobia.

#### 2.5.1. Personal Information Form

The investigator-developed form contained 13 items capturing demographic and clinical background data. Domains covered included: age and sex; anthropometric variables (height, weight, BMI); social variables (marital, employment, and income status; educational attainment); and health-related history (chronic illness, prior surgical procedures, current medications, tobacco and alcohol use, and familial cardiac disease) [13,14].

#### 2.5.2. Intensive Care Units Mobility Scale

Patients’ achieved level of physical activity in the ICU was rated using the Intensive Care Units Mobility Scale. Originally introduced by Hodgson and colleagues in 2014, this single-domain observational instrument scores the maximum daily mobility milestone reached by the patient on an 11-point ordinal scale (0–10) [15]. The Turkish validity and reliability study of the scale was conducted by İbrahimoğlu, Saray Kılıç, and Açıkgöz (2022), who reported a Kappa value of 0.84, indicating high test–retest reliability [16]. In the present study, the IMS was administered during the patient’s first postoperative mobilization session by the bedside nurse who supervised that session, in accordance with the original validation protocol [15,16]. To minimize observer bias, the nurse who scored the IMS performed only the observational rating and was not involved in administering the Tampa Scale of Kinesiophobia at that moment. Inter-observer reliability of the IMS was not formally re-tested in the present sample; we relied on the previously established Turkish validation [16] (Kappa = 0.84). This is acknowledged as a limitation. It should also be noted that the observed IMS scores in this sample were restricted to the 4–8 range out of the possible 0–10, suggesting a ceiling/floor effect that may attenuate observed correlations and reduce subgroup variance.

### 2.6. Tampa Scale of Kinesiophobia

Patient-reported fear of movement was assessed with the Tampa Scale of Kinesiophobia, a self-administered questionnaire originally constructed by Miller, Kori, and Todd in 1991 and subsequently refined by Vlaeyen and colleagues in 1995 [17,18]. The Turkish adaptation, validity, and reliability study was conducted by Tunca Yılmaz, Yakut, Uygur, and Uluğ (2011) [19]. The Cronbach’s alpha coefficient of the scale was reported as 0.80, indicating high internal consistency. The instrument contains 17 statements rated on a four-point Likert scale (1 = strongly disagree to 4 = strongly agree), tapping into three conceptual domains: avoidance of physical exertion, perceived functional limitation, and apprehension of re-injury. Higher scores indicate higher levels of kinesiophobia. In the present study, the Cronbach’s alpha coefficient of the Tampa Scale of Kinesiophobia was found to be 0.81, confirming the reliability of the scale.

### 2.7. Data Collection Procedure

Data acquisition took place from December 2024 through April 2025 via in-person bedside interviews. Eligible participants were enrolled while undergoing postoperative care at the same two ICU sites described above. All sessions were conducted under the supervision of a nurse, and visual/auditory privacy was maintained throughout each interview. Individual interviews lasted approximately 20–25 min. The study was conducted during patients’ first mobilization session, which lasted an average of 40–45 min. The Personal Information Form, Intensive Care Units Mobility Scale, and Tampa Scale of Kinesiophobia were used for data collection. The Tampa Scale of Kinesiophobia was administered during the first planned mobilization session after surgery, only when patients were hemodynamically stable, fully conscious, extubated, and able to communicate effectively. Prior to data collection, patients’ cognitive orientation was clinically assessed by the nurse, and individuals receiving continuous sedative medication or showing signs of confusion were not included. All data were collected under nurse supervision to ensure patient safety and accurate understanding of the scale items.

Considerations regarding measurement timing and potential bias: The administration of the Tampa Scale of Kinesiophobia during the first postoperative mobilization session in the ICU represents a clinically meaningful but potentially bias-prone time point. Three sources of measurement bias are acknowledged. First, postoperative pain may transiently elevate kinesiophobia scores; in the present study, pain intensity (e.g., VAS or NRS) was not formally measured and was therefore not used as a covariate. Second, residual sedation may impair self-report accuracy; in the present study, sedation status was assessed clinically by the bedside nurse rather than with a standardized instrument such as the Richmond Agitation–Sedation Scale (RASS), and only patients judged to be alert, fully conscious, and able to communicate effectively were enrolled. Third, postoperative delirium may also affect self-report; delirium was not formally screened with validated tools (e.g., the Confusion Assessment Method for the ICU, CAM-ICU), although patients showing signs of confusion were excluded based on clinical judgment. The absence of formal measurement of pain, sedation level, and delirium is acknowledged as a methodological limitation, and these variables were not entered as covariates in the analyses; this is discussed further in the Limitations.

### 2.8. Ethical Considerations

This study was conducted in accordance with the principles of the Declaration of Helsinki (1975, revised in 2013). Ethical approval for the study was obtained from the Clinical Research Ethics Committee of Kartal Dr. Lütfi Kırdar City Hospital (Istanbul, Türkiye) on 5 November 2024 (Approval No: 2024/19/59).

In addition, institutional permissions were obtained from the Chief Physician of Koşuyolu High Specialty Training and Research Hospital (Permission No: E-53838792-604.01-260582843) and from the Chief Physician of Maltepe University Medical Faculty Training and Research Hospital (Permission No: E-32835138-108.01-333471).

### 2.9. Informed Consent Statement

Written informed consent was obtained from all patients involved in the study prior to participation.

### 2.10. Data Analysis

The research data were analyzed using SPSS version 25.0 (IBM Corp., Armonk, NY, USA). Descriptive statistics included frequency, percentage, mean, standard deviation, minimum, and maximum values. Continuous variables were summarized as mean ± SD; categorical variables as n (%).

Normality of continuous variables was assessed using the Shapiro–Wilk test in addition to skewness and kurtosis values; absolute skewness ≤ 2 and absolute kurtosis ≤ 7 were considered acceptable for assuming approximate normality. The overall distributions of the Tampa Scale of Kinesiophobia (skewness = −0.13; kurtosis = 1.29) and the Intensive Care Units Mobility Scale (skewness = 0.49; kurtosis = 0.71) were within these limits, supporting approximate normality at the full-sample level.

The independent samples *t*-test was used for parametric variables, while the Mann–Whitney U and Kruskal–Wallis H tests were used for non-parametric variables. Pearson correlation analyses were applied to examine association between variables. Although the overall distributions of the scale scores were approximately normal, non-parametric tests were preferred for subgroup comparisons because some subgroups had small sample sizes and unequal group distributions, which could violate the assumptions of parametric tests. Specifically, the Mann–Whitney U test was used for two-group comparisons (Gender, Marital status, Employment, Chronic disease, History of surgery, Regular medication use, Smoking, Alcohol use, Family history of chronic disease), and the Kruskal–Wallis H test was used for comparisons across more than two groups (Age, Height, Weight). The Pearson correlation coefficient was used to assess the association between the Tampa and IMS total scores at the full-sample level, and the 95% confidence interval (CI) for r was calculated using Fisher’s z-transformation.

Effect sizes were reported alongside *p*-values to support interpretation of clinical relevance: r = |Z|/√N for Mann–Whitney U comparisons (small ≈ 0.10, medium ≈ 0.30, large ≈ 0.50); η^2^ = (H − k + 1)/(N − k) for Kruskal–Wallis comparisons (small ≈ 0.01, medium ≈ 0.06, large ≈ 0.14); r^2^ for Pearson correlation; and standardized β with 95% CI for regression coefficients. All *p*-values were two-tailed.

To address the issue of multiple subgroup comparisons, the Bonferroni correction was formally applied. Within each outcome (kinesiophobia and mobilization), k = 12 subgroup comparisons were performed; therefore, the corrected significance threshold was set at α_adj = 0.05/12 = 0.00417. Both uncorrected and Bonferroni-adjusted *p*-values (*p*_adj = *p* × k, capped at 1.000) are reported in table. Uncorrected *p* < 0.05 was considered nominally significant, whereas *p*_adj < 0.05 was considered confirmatory.

Multivariable analysis: To address the possibility that the bivariate association between kinesiophobia and mobilization was confounded by sociodemographic or clinical factors, a multivariable linear regression analysis was conducted with the IMS total score as the dependent variable. Independent variables were selected on the basis of the univariate subgroup analyses that showed nominally significant associations with mobilization (sex, smoking) and with kinesiophobia (history of surgery), as well as age as a clinically relevant continuous covariate, and the Tampa total score as the primary predictor. Categorical variables were entered as dummy variables (Sex: 0 = Male, 1 = Female; Smoking: 0 = No, 1 = Yes; History of surgery: 0 = No, 1 = Yes). Multicollinearity was assessed using the variance inflation factor (VIF), with VIF < 5 considered acceptable. Residual normality and homoscedasticity were inspected via standardized residual plots, and influential observations were screened using Cook’s distance (threshold > 1).

## 3. Results

The sociodemographic and clinical background characteristics of the study cohort, captured via the Personal Information Form, are summarized in Table 1.

The cohort had a mean age of 58.30 ± 6.50 years (SD), with nearly half (45.8%) aged ≥62 years; men predominated, accounting for 76.0% of participants. Mean BMI was 27.53 ± 5.84 kg/m^2^ (SD); 64.6% fell within the overweight category and 19.8% met criteria for obesity. Mean height and body weight were 172.85 ± 7.31 cm (SD) and 82.40 ± 7.91 kg (SD), respectively (Table 1).

Most participants were married (89.6%) and not currently working (51.0%). High school graduation represented the most common educational attainment (30.2%). With respect to financial status, 80.2% indicated that household income matched expenses (Table 1).

A chronic medical condition was reported by 83.3% of participants; cardiovascular conditions (39.6%), diabetes mellitus (29.2%), and hypertension (21.9%) were the most frequent. Roughly half (51.0%) had undergone prior surgery, and 62.5% used medications on a regular basis. Tobacco use was noted in 38.5% and alcohol use in 3.1%, while a familial history of chronic disease was disclosed by 59.4% (Table 1).

Internal consistency of the TSK in the present sample was strong (Cronbach’s α = 0.817), confirming acceptable psychometric performance of the instrument for assessing movement-related fear in this clinical context (Table 2).

The mean item-level score was 2.75 ± 0.38, and the total score averaged 46.81 ± 6.51 (observed range: 27–62; theoretical range: 17–68). Distributional indices (skewness = −0.13; kurtosis = 1.29) fell within accepted thresholds (|skewness| ≤ 2; |kurtosis| ≤ 7), supporting approximate normality. Collectively, these descriptors point to fear-of-movement levels in the low-to-moderate range (Table 2).

The IMS is a single-item, 11-point observation-based scale (range 0–10), and Cronbach’s alpha was therefore not calculated. The sample mean was 5.48 ± 0.73, with all observations falling between 4 and 8. Skewness (0.49) and kurtosis (0.71) indicated near-normality. However, the observed IMS range (4–8) covered only the upper-middle portion of the theoretical 0–10 range, indicating a restricted distribution and a potential ceiling/floor effect that may attenuate true correlations and limit subgroup variance (Table 2).

Subgroup analyses contrasting kinesiophobia and mobilization scores across demographic and clinical strata are presented in Table 3. Effect sizes for two-group comparisons were calculated as r = |Z|/√N (Mann–Whitney U test); η^2^ was calculated for Kruskal–Wallis H tests; and 95% confidence intervals (CIs) for r were derived using Fisher’s z-transformation. To account for multiple subgroup testing, the Bonferroni correction was applied for k = 12 subgroup comparisons within each outcome (corrected α = 0.05/12 = 0.00417), and Bonferroni-adjusted *p*-values (*p*_adj) are reported alongside uncorrected *p*-values (Table 3).

At the uncorrected threshold, female participants showed higher IMS scores than males (*p* = 0.046, r = 0.20, 95% CI: 0.00–0.39). Among married individuals, TSK scores ran higher than among single participants (*p* = 0.034, r = 0.22, 95% CI: 0.02–0.40). Patients reporting a prior surgical history had lower kinesiophobia values (*p* = 0.020, r = 0.24, 95% CI: 0.04–0.42) (Table 3).

Non-smokers achieved higher mobilization scores than current smokers (*p* = 0.012, r = 0.26, 95% CI: 0.06–0.44). Likewise, those with a familial history of chronic disease scored lower on the TSK (*p* = 0.014, r = 0.25, 95% CI: 0.05–0.43). However, after Bonferroni correction for multiple subgroup comparisons, none of these differences remained statistically significant (all *p*_adj > 0.10), and they should therefore be interpreted as exploratory rather than confirmatory. The remaining variables—age, height, weight, education, income, chronic-disease history, regular medication use, and alcohol consumption—yielded no significant differences in either outcome (all uncorrected *p* > 0.05; all *p*_adj = 1.000) (Table 3).

Consistent with the study’s primary aim, the bivariate Pearson correlation between TSK and IMS total scores was examined. The analysis yielded a small inverse association that reached the conventional significance threshold (r = −0.104, 95% CI: −0.298 to 0.099, r^2^ = 0.011, *p* = 0.041), with kinesiophobia explaining roughly 1.1% of the variance in IMS scores. Because the shared variance is minimal and the upper bound of the 95% CI for r crosses zero, the clinical relevance of this association should be interpreted with caution and should not be regarded as evidence of a meaningful effect (Table 4).

To address potential confounding by clinical and demographic factors that showed nominally significant univariate associations (Table 3)—namely sex, age, smoking status, and history of surgery—a multivariable linear regression analysis was conducted with the IMS total score as the dependent variable and the TSK total score as the primary predictor. After adjustment, kinesiophobia did not remain a statistically significant predictor of mobilization (β = −0.092, *p* = 0.360), whereas smoking (β = −0.279, *p* = 0.008) and female sex (β = 0.215, *p* = 0.039) emerged as the strongest independent predictors. The full model accounted for 15.1% of the variance in mobilization scores (R^2^ = 0.151; adjusted R^2^ = 0.104; F(5, 90) = 3.198; *p* = 0.011), with all variance inflation factor (VIF) values below 1.2, indicating no concerning multicollinearity (Table 5).

## 4. Discussion

In this study the cross-sectional association between kinesiophobia and mobilization level in patients who underwent open-heart surgery was examined, and a weak, statistically significant negative association was identified between kinesiophobia and mobilization. However, after adjustment for sociodemographic and clinical covariates in the multivariable regression model (Table 5), kinesiophobia was no longer an independent predictor of mobilization, and the bivariate effect was largely attributable to confounding by smoking status and sex. The findings indicate that mobilization in the postoperative period may relate not only to physiological adequacy but also to patients’ perceptions and fears regarding movement. Particularly during the intensive care process, individual characteristics, health history, and psychosocial factors associated with mobilization may relate to the course of recovery, although a cross-sectional design does not allow inferences about directionality or causality. Kinesiophobia may be considered as one of several psychosocial factors potentially associated with patients’ participation in postoperative mobilization.

The sample profile largely mirrored that reported for cardiac surgery cohorts elsewhere. Older adults predominated, in line with prior literature [7]. Comorbidity burden was substantial, with most participants carrying at least one chronic illness, prior surgical exposure, tobacco use, or familial chronic-disease history [20,21]. Male preponderance in our cohort is consistent with the well-documented sex disparity in cardiovascular morbidity [22]. Likewise, the high frequency of overweight status echoes patterns previously reported in comparable surgical populations [23]. A majority being married is a frequently noted characteristic of this surgical population [24], and educational attainment skewed toward low-to-moderate levels—also a common observation [25]. The substantial proportion of participants outside active employment has been documented in earlier work [4], and the predominantly balanced income–expense profile aligns with previously described socioeconomic patterns in this population [26]. Taken together, these characteristics support that the present cohort reflects a representative cardiac-surgery profile and that subsequent comparisons with the literature are appropriate.

When kinesiophobia and mobilization levels were evaluated according to sociodemographic characteristics in this study, several variables showed nominally significant differences at the uncorrected level, although none of these differences remained statistically significant after Bonferroni correction for multiple comparisons (all *p*_adj > 0.10; Table 3). These subgroup findings should therefore be interpreted as exploratory rather than confirmatory. Across age strata, kinesiophobia displayed an upward trend and mobilization a downward trend; however, neither pattern reached statistical significance, indicating that age in isolation cannot be regarded as a determining factor [27]. While no significant association was found between education or income level and kinesiophobia or mobilization, the literature emphasizes that these variables may have indirect and contextual effects [28,29]. Although kinesiophobia levels were similar between genders, higher mobilization levels were observed in female patients at the uncorrected level (*p* = 0.046, r = 0.20); this finding did not survive Bonferroni correction (*p*_adj = 0.552), and underlying reasons cannot be inferred from a cross-sectional design [30].

Regarding marital status, higher kinesiophobia levels in married patients (uncorrected *p* = 0.034; *p*_adj = 0.408) may be associated with family responsibilities and concerns about re-illness [24]. This association is preliminary and requires confirmation in adequately powered studies. No significant difference was found between employment status and kinesiophobia or mobilization, and this association may vary depending on individual and environmental factors [31]. The lack of a significant difference for chronic disease history may indicate that individuals accustomed to regular medical follow-up can manage their physical conditions more effectively. Conversely, higher kinesiophobia levels in patients without a prior surgical history (uncorrected *p* = 0.020; *p*_adj = 0.240) may be associated with the absence of prior surgical experience and could highlight the potential relevance of individualized nursing approaches [32]. Lower mobilization levels observed in smokers were one of the strongest subgroup signals in this study (uncorrected *p* = 0.012; *p*_adj = 0.144) and persisted as an independent predictor in the multivariable model (β = −0.279, *p* = 0.008; Table 5), which is consistent with the well-documented limiting effects of smoking on physical performance [33]. Although no significant difference was found in terms of alcohol use, higher kinesiophobia levels in individuals with a family history of heart disease (uncorrected *p* = 0.014; *p*_adj = 0.168) may be associated with negative observational experiences and family-related health concerns; this finding should be interpreted cautiously and is not supported after correction for multiple testing [18]. Overall, these findings suggest that individual characteristics such as gender, marital status, smoking, and surgical experience may be associated with kinesiophobia and mobilization levels, although the cross-sectional design does not permit causal interpretation. They may inform a holistic evaluation of patients’ psychosocial characteristics in postoperative care.

The bivariate analysis yielded a weak inverse association between TSK and IMS scores in this open-heart surgery cohort: higher fear-of-movement values corresponded to slightly lower mobilization scores, although the magnitude of this signal was modest (r = −0.104; r^2^ = 0.011), and the 95% CI for r (−0.298 to 0.099) crossed zero on its upper bound. After multivariable adjustment, this association was no longer statistically significant (β = −0.092; *p* = 0.360; Table 5), indicating that the bivariate correlation may have been confounded by smoking status and sex. Conceptually, kinesiophobia may operate as one psychological component shaping how patients engage with their post-surgical bodies and could plausibly contribute to avoidance behaviors when intersecting with concurrent factors such as pain experience, apprehension about the surgical wound, and worry about recurrent illness. It is therefore best framed as one element within a broader set of factors that may relate to reduced mobilization activity early after surgery.

The existing literature has reported associations between kinesiophobia and mobilization across various clinical populations [32,34,35,36]. In contrast to the present study’s findings of a weak association in cardiac surgery patients, Luque-Suárez et al. (2019) reported that increased kinesiophobia was closely associated with decreased mobilization in individuals with chronic musculoskeletal pain [37]. Similarly, Özdemir et al. (2024) reported that as difficulties experienced during the mobilization process increased, kinesiophobia levels also increased, and a positive moderate association was found between these two variables in patients undergoing total knee arthroplasty [38]. Other studies have likewise emphasized the reciprocal interaction between mobilization and kinesiophobia [9,13,38]. The discrepancy between these moderate associations reported in non-cardiac populations and the weak association observed in our cardiac surgery sample may reflect differences in patient populations, measurement timing (early ICU period vs. later rehabilitation phases), the restricted variance of IMS scores in our sample (range 4–8 of 0–10), and unmeasured confounders specific to the early postoperative cardiac setting. Furthermore, kinesiophobia has been linked in earlier work to an individual’s prior history of physical activity, the way pain is appraised cognitively, and broader psychosocial context—elements that could plausibly influence engagement in mobilization efforts and the trajectory of physical recovery in the postoperative window [11,39]. Singh et al. (2026) demonstrated that high levels of kinesiophobia were associated with delayed first mobilization attempts and longer hospital length of stay [40]. These findings indicate that kinesiophobia may be associated with mobilization behavior; however, its effect size is small and should be interpreted with caution. A systematic assessment of patients’ kinesiophobia levels alongside attention to other relevant factors (e.g., smoking, sex, pain, sedation) may be considered when planning early mobilization practices, although the present study does not establish kinesiophobia as a primary determinant of mobilization [27,41].

Statistical significance versus clinical relevance. Although the observed correlation between kinesiophobia and mobilization reached statistical significance (r = −0.104, *p* = 0.041), the magnitude of the effect was small (r^2^ ≈ 0.011), indicating that kinesiophobia accounted for only approximately 1% of the variance in mobilization scores. This raises important questions about the clinical significance of the finding. A statistically significant result in a sample of this size may reflect a true but minor effect, the influence of unmeasured confounders, or a chance finding (Type I error), particularly considering that the *p*-value was very close to the 0.05 threshold and the 95% CI for r approached zero. Furthermore, in the multivariable regression model (Table 5), kinesiophobia did not remain a statistically significant predictor of mobilization once sex, smoking, age, and surgical history were controlled for. Therefore, while kinesiophobia may merit clinical attention as one of several psychosocial factors during postoperative care, it should not be considered a primary determinant of mobilization outcomes based on this study alone.

Confounding and unmeasured variables. This study did not measure or adjust for several variables known to be associated with early postoperative mobilization, including pain intensity (e.g., VAS or NRS), level of sedation (e.g., RASS), incidence of postoperative delirium (e.g., CAM-ICU), type and complexity of cardiac surgery (CABG vs. valve vs. combined procedures), duration of mechanical ventilation, length of cardiopulmonary bypass, and the postoperative analgesic regimen. In the multivariable model presented (Table 5), only a limited set of sociodemographic and clinical covariates (sex, age, smoking, history of surgery) could be included. Residual confounding from the unmeasured factors listed above may attenuate or amplify the observed association between kinesiophobia and mobilization. The same residual confounding may also explain the weak magnitude of the bivariate correlation. Future studies should incorporate these clinical variables as covariates in adjusted models and use longitudinal designs that permit clearer separation of psychological and physiological determinants.

Several exploratory subgroup analyses were undertaken in the present study to probe potential differences along sociodemographic and clinical lines; nonetheless, these results warrant cautious interpretation. Performing multiple comparisons increases the risk of Type I error, and after Bonferroni correction (k = 12 comparisons within each outcome; corrected α = 0.00417), none of the subgroup-level differences originally observed at the uncorrected level remained statistically significant. In addition, the correlation between kinesiophobia and mobilization level was statistically significant but weak, and was not retained as an independent predictor in the multivariable model, suggesting that mobilization behavior in the postoperative period is shaped by multiple interacting physiological, psychological, and environmental factors rather than by kinesiophobia alone. Future studies with larger samples, longitudinal designs, and more controlled analytical models are needed to further clarify the determinants of mobilization in patients undergoing open-heart surgery.

### Limitations

A number of methodological constraints warrant acknowledgment. First, the cross-sectional design precludes any inference of temporality or causality between kinesiophobia and mobilization; the design permits only the identification of associations at a single time point. Second, the observed correlation was statistically significant but small in magnitude (r = −0.104, r^2^ ≈ 0.011), and the study was a priori powered to detect a medium effect size (d = 0.50, equivalent to |r| ≈ 0.24); thus, the present sample is underpowered to confirm an effect of the magnitude actually observed. Detecting a correlation of |r| = 0.10 with 80% power and α = 0.05 would require approximately 783 participants. Third, the IMS scores were restricted to a 4–8 range out of the possible 0–10, suggesting a ceiling/floor effect that limits variance and may attenuate observed correlations and subgroup differences. Fourth, several variables that may be associated with early mobilization—including pain intensity, sedation level, postoperative delirium, type of surgery, duration of mechanical ventilation, and analgesic regimen—were not formally measured and were not entered as covariates in the analyses. Fifth, multiple subgroup comparisons were performed, increasing the risk of Type I error; while the Bonferroni correction was formally applied, the uncorrected subgroup-level differences should be interpreted as exploratory rather than confirmatory. Sixth, inter-rater reliability of the IMS administration was not formally re-tested in the present sample, and observers were not blinded to participants’ Tampa scores, which may introduce observer bias. Seventh, delivering the Tampa Scale as a self-report questionnaire in the immediate postoperative ICU window leaves the resulting scores susceptible to short-term influences from acute pain, residual sedation, or postoperative anxiety, all of which constitute potential sources of measurement bias. Finally, enrollment was confined to two Istanbul centers via consecutive sampling, which may constrain external validity across other regions, institutional contexts, and cardiac surgical populations. Despite these limitations, the present analyses offer preliminary indications that kinesiophobia may operate as one of several psychosocial factors with a possible link to early postoperative mobilization following open-heart surgery, while smoking and sex stand out as more robust independent correlates of mobilization within this cohort.

## 5. Conclusions

This cross-sectional study identified a weak, statistically significant negative association between kinesiophobia and mobilization level in patients undergoing open-heart surgery (r = −0.104, *p* = 0.041). However, this association did not remain statistically significant after multivariable adjustment for sex, age, smoking status, and history of surgery (β = −0.092, *p* = 0.360), and kinesiophobia accounted for only approximately 1% of the variance in mobilization scores (r^2^ = 0.011). The clinical relevance of this finding therefore appears limited. Mobilization emerged as a multidimensional construct, potentially relating not only to physical readiness but also to how patients interpret and respond to movement, alongside clinical variables such as smoking and sex, which functioned as more robust independent correlates of mobilization in this dataset. Nurses involved in postoperative cardiac care may benefit from awareness of psychological barriers such as kinesiophobia and from considering individualized, progressive, and safety-oriented mobilization plans; however, given the weak effect size and the cross-sectional design, mobilization planning should not rest on kinesiophobia as a single or primary factor. In conclusion, kinesiophobia may be considered as one of several psychosocial factors potentially associated with mobilization in the early postoperative period following open-heart surgery, rather than as a primary determinant of mobilization outcomes. Future longitudinal studies with larger samples, multivariable adjustment for clinical confounders such as pain, sedation, delirium, and surgical complexity, and standardized timing of measurements are needed to clarify the role of kinesiophobia in postoperative recovery.

## Figures and Tables

**Table 1:** Sociodemographic and Health History Characteristics of the Patients (n = 96).

Part A. Continuous variables—Mean ± SD (range).			
Variable	n	Mean ± SD	Range
Age (years)	96	58.30 ± 6.50	22–80
Body Mass Index (kg/m^2^)	96	27.53 ± 5.84	18.5–38.6
Height (cm)	96	172.85 ± 7.31	158–190
Weight (kg)	96	82.40 ± 7.91	62–112
Part B. Categorical variables—n (%)			
Variable		Category	n (%)
Age group		22–41 years	10 (10.4)
		42–61 years	42 (43.8)
		≥62 years	44 (45.8)
Gender		Female	23 (24.0)
		Male	73 (76.0)
BMI category		Normal (18.5–24.9)	15 (15.6)
		Overweight (25.0–29.9)	62 (64.6)
		Obese (≥30.0)	19 (19.8)
Height category		<165 cm	18 (18.8)
		165–175 cm	38 (39.6)
		>175 cm	40 (41.7)
Weight category		Underweight–slightly overweight	5 (5.2)
		Normal weight	39 (40.6)
		Overweight	37 (38.5)
		Obese	15 (15.6)
Marital status		Married	86 (89.6)
		Single	10 (10.4)
Education level		Illiterate	4 (4.2)
		Literate/Primary school	27 (28.1)
		Middle school	27 (28.1)
		High school	29 (30.2)
		University	9 (9.4)
Employment status		Employed	47 (49.0)
		Unemployed	49 (51.0)
Income level		Income < expenses	6 (6.3)
		Income = expenses	77 (80.2)
		Income > expenses	13 (13.5)
Chronic disease		Yes	80 (83.3)
		No	16 (16.7)
Type of chronic disease		None	9 (9.4)
		Hypertension	21 (21.9)
		Diabetes mellitus	28 (29.2)
		Cardiovascular disease	38 (39.6)
History of surgery		Yes	49 (51.0)
		No	47 (49.0)
Regular medication use		Yes	60 (62.5)
		No	36 (37.5)
Smoking status		Yes	37 (38.5)
		No	59 (61.5)
Daily cigarette consumption (among smokers, n = 37)		1–5 cigarettes	4 (10.8)
		6–10 cigarettes	22 (59.5)
		11–15 cigarettes	7 (18.9)
		16–20 cigarettes	4 (10.8)
Alcohol use		Yes	3 (3.1)
		No	93 (96.9)
Family history of chronic disease		Yes	57 (59.4)
		No	39 (40.6)

Abbreviations: BMI, body mass index; n, number of patients; SD, standard deviation. Notes: Continuous variables are presented as Mean ± SD; categorical variables as n (%). For “Daily cigarette consumption,” percentages are recalculated within smokers (n = 37) for clarity.

**Table 2:** Descriptive Statistics, Normality, and Reliability Results for the Measurement Instruments.

Variables	n	Item Mean ± SD	Total Score (Mean ± SD)	Min	Max	Theoretical Range	Skewness	Kurtosis	Cronbach’s α
Tampa Scale of Kinesiophobia	96	2.75 ± 0.38	46.81 ± 6.51	27	62	17–68	−0.13	1.29	0.817
ICU Mobility Scale	96	-	5.48 ± 0.73	4.0	8.0	0–10	0.49	0.71	- ^a^

Abbreviations: SD, standard deviation; α, Cronbach’s alpha; n, number of patients. Note: ^a^ Cronbach’s α was not calculated for the IMS because it is a single-item, observation-based scale.

**Table 3:** Distribution of TSK and IMS Scores by Sociodemographic and Clinical Characteristics (n = 96).

Part A—Kinesiophobia (TSK total score)									
Variable	Group	n	Mean ± SD	Test	Statistic	Effect size	95% CI for r	*p* (uncorr.)	*p*_adj
Gender	Female	23	48.81 ± 1.73	MWU	Z = −0.956	r = 0.10	(−0.11, 0.30)	0.339	1.000
	Male	73	47.00 ± 4.27						
Marital status	Married	86	50.03 ± 4.36	MWU	Z = −2.116	r = 0.22	(0.02, 0.40)	0.034	0.408
	Single	10	39.35 ± 1.11						
Employment	Employed	47	47.65 ± 3.70	MWU	Z = −0.515	r = 0.05	(−0.15, 0.25)	0.607	1.000
	Unemployed	49	48.25 ± 2.73						
Chronic disease	Yes	80	48.05 ± 4.39	MWU	Z = −0.355	r = 0.04	(−0.16, 0.24)	0.722	1.000
	No	16	50.75 ± 1.56						
History of surgery	Yes	49	42.06 ± 2.45	MWU	Z = −2.321	r = 0.24	(0.04, 0.42)	0.020	0.240
	No	47	55.21 ± 3.77						
Regular medication	Yes	60	46.60 ± 4.10	MWU	Z = −0.866	r = 0.09	(−0.11, 0.29)	0.387	1.000
	No	36	51.67 ± 2.16						
Smoking	Yes	37	49.74 ± 2.49	MWU	Z = −0.255	r = 0.03	(−0.18, 0.23)	0.799	1.000
	No	59	47.72 ± 3.92						
Alcohol use	Yes	3	42.83 ± 0.45	MWU	Z = −0.359	r = 0.04	— ^a^	0.719	1.000
	No	93	48.68 ± 4.63						
Family history of CD	Yes	57	43.36 ± 3.10	MWU	Z = −2.456	r = 0.25	(0.05, 0.43)	0.014	0.168
	No	39	56.01 ± 3.31						
Age (years)	22–41	10	35.45 ± 0.76	KW	H = 2.544	η^2^ = 0.006	— ^b^	0.280	1.000
	42–61	42	49.17 ± 2.66						
	≥62	44	50.83 ± 3.58						
Height (cm)	≤165	18	2.88 ± 1.40 ^c^	KW	H = 1.755	η^2^ ≈ 0	—	0.416	1.000
	165–175	38	2.70 ± 2.34						
	≥175	40	2.84 ± 3.67						
Weight (BMI)	Underweight	5	2.52 ± 0.72 ^c^	KW	H = 2.966	η^2^ ≈ 0	—	0.227	1.000
	Normal	39	2.75 ± 2.25						
	Overweight	37	2.82 ± 2.92						
	Obese	15	2.90 ± 2.71						
Part B—Mobilization (IMS total score)									
Variable	Group	n	Mean ± SD	Test	Statistic	Effect size	95% CI for r	*p* (uncorr.)	*p*_adj
Gender	Female	23	5.08 ± 3.31	MWU	Z = −2.000	r = 0.20	(0.00, 0.39)	0.046	0.552
	Male	73	4.56 ± 6.15						
Marital status	Married	86	4.63 ± 5.75	MWU	Z = −1.354	r = 0.14	(−0.07, 0.33)	0.176	1.000
	Single	10	5.43 ± 4.08						
Employment	Employed	47	4.74 ± 5.69	MWU	Z = −0.372	r = 0.04	(−0.16, 0.24)	0.710	1.000
	Unemployed	49	4.92 ± 4.27						
Chronic disease	Yes	80	4.76 ± 6.80	MWU	Z = −0.765	r = 0.08	(−0.13, 0.28)	0.444	1.000
	No	16	5.29 ± 2.04						
History of surgery	Yes	49	4.77 ± 4.97	MWU	Z = −0.316	r = 0.03	(−0.17, 0.23)	0.752	1.000
	No	47	4.93 ± 5.14						
Regular medication	Yes	60	5.22 ± 5.96	MWU	Z = −1.859	r = 0.19	(−0.01, 0.38)	0.063	0.756
	No	36	4.23 ± 3.72						
Smoking	Yes	37	4.01 ± 4.86	MWU	Z = −2.521	r = 0.26	(0.06, 0.44)	0.012	0.144
	No	59	5.38 ± 4.92						
Alcohol use	Yes	3	4.31 ± 1.00	MWU	Z = −0.372	r = 0.04	— ^a^	0.710	1.000
	No	93	4.87 ± 7.04						
Family history of CD	Yes	57	5.02 ± 4.98	MWU	Z = −0.729	r = 0.07	(−0.13, 0.27)	0.466	1.000
	No	39	4.60 ± 5.12						
Age (years)	22–41	10	5.91 ± 3.13	KW	H = 4.228	η^2^ = 0.024	—	0.121	1.000
	42–61	42	5.15 ± 4.99						
	≥62	44	4.33 ± 3.71						
Height (cm)	≤165	18	5.72 ± 3.52	KW	H = 2.856	η^2^ = 0.009	—	0.240	1.000
	165–175	38	5.37 ± 3.88						
	≥175	40	5.48 ± 4.74						
Weight (BMI)	Underweight	5	6.00 ± 2.24	KW	H = 3.282	η^2^ = 0.003	—	0.194	1.000
	Normal	39	5.54 ± 4.74						
	Overweight	37	5.30 ± 4.26						
	Obese	15	5.60 ± 1.97						

Abbreviations: CD, chronic disease; CI, confidence interval; IMS, Intensive Care Units Mobility Scale; KW, Kruskal–Wallis H test; MWU, Mann–Whitney U test; n, sample size; *p*_adj, Bonferroni-adjusted *p*-value; r, effect size for Mann–Whitney U test; TSK, Tampa Scale of Kinesiophobia; η^2^, eta-squared. Footnotes: ^a^ The 95% CI was not reported for alcohol use because of marked group imbalance. ^b^ The 95% CI for r is not applicable to KW comparisons; η^2^ is reported instead. ^c^ Effect sizes were calculated as r = |Z|/√N for MWU tests and η^2^ = (H − k + 1)/(N − k) for KW tests. adjusted *p*-values were capped at 1.000. After Bonferroni correction, no subgroup comparison remained statistically significant.

**Table 4:** Correlation Between Kinesiophobia and Mobilization Level (n = 96).

Variable 1	Variable 2	r	r^2^	95% CI for r	*p*
TSK (total score)	IMS (total score)	−0.104	0.011	(−0.298, 0.099)	0.041

Abbreviations: CI, confidence interval; IMS, Intensive Care Units Mobility Scale; r, Pearson correlation coefficient; r^2^, coefficient of determination; TSK, Tampa Scale of Kinesiophobia. Notes: Pearson correlation analysis was used. The 95% CI for r was calculated using Fisher’s z-transformation. A weak negative correlation was found between TSK and IMS scores. TSK scores explained approximately 1.1% of the variance in IMS scores. Although the correlation reached statistical significance, the magnitude of the association was small; therefore, the result should be interpreted with caution.

**Table 5:** Multivariable Linear Regression Predicting IMS Total Score (n = 96).

Predictor	B	SE(B)	β (Standardized)	95% CI for B	t	*p*	VIF
(Constant)	6.812	0.928	—	(4.969, 8.655)	7.341	<0.001	—
TSK total score	−0.0103	0.0112	−0.092	(−0.0325, 0.0119)	−0.920	0.360	1.124
Sex (Female = 1) ^a^	0.365	0.174	0.215	(0.019, 0.711)	2.098	0.039	1.063
Age (years)	−0.0185	0.0114	−0.165	(−0.0411, 0.0041)	−1.623	0.108	1.135
Smoking (Yes = 1) ^a^	−0.418	0.154	−0.279	(−0.724, −0.112)	−2.714	0.008	1.045
History of surgery (Yes = 1) ^a^	−0.066	0.150	−0.045	(−0.364, 0.232)	−0.440	0.661	1.052
Model fit							
R	0.389						
R^2^	0.151						
Adjusted R^2^	0.104						
F (5, 90)	3.198					0.011	

Abbreviations: B, unstandardized regression coefficient; CI, confidence interval; IMS, Intensive Care Units Mobility Scale; SE, standard error; TSK, Tampa Scale of Kinesiophobia; VIF, variance inflation factor; β, standardized regression coefficient. Note: ^a^ Reference categories were as follows: sex, male; smoking, no; history of surgery, no. Smoking was independently associated with lower IMS scores, whereas female sex was associated with higher IMS scores. The model explained 15.1% of the variance in IMS total score.

## Data Availability

The data presented in this study are available on request from the corresponding author. The data are not publicly available due to ethical restrictions related to patient privacy and the conditions of the institutional review board’s approval (Clinical Research Ethics Committee of Kartal Dr. Lütfi Kırdar City Hospital, Approval No: 2024/19/59).
